# Supplementary material for: Limited Genetic Diversity of blaCMY-2-Containing IncI1-pST12 Plasmids from Enterobacteriaceae of Human and Broiler Chicken Origin in The Netherlands
Source: Microorganisms. 2020 Nov 8;8(11):1755. doi: 10.3390/microorganisms8111755 (PMC7695270; doi:10.3390/microorganisms8111755)
Supplement: Supplementary file 1 [file microorganisms-08-01755-s001.pdf]

Table S1. Summary of screened cases and sequenced and selected isolates

Collection	Total number of screened cases		Number of sequenced isolates based on AmpC phenotype		N of isolates containing IncI1-pST12 replicon and *bla*$_{CMY-2}$ gene		N of isolates included in study	
	Human	Broiler	Human	Broiler	Human	Broiler	Human	Broiler
I-4-1 Health study	380	119	19	22	0	11	0	9
Amphia prevalence screening 2017	378	0	14	0	2	0	2	0
E. coli blood cultures 2013-2015	1749	0	51	0	2	0	2	0
Salmonella enteritidis fecal culture	1	0	1	0	1	0	1	0
Total	2508	119	85	22	5	11	5	9

Table S2. Distance matrix containing number of allele differences between the included *E. coli* isolates based on wgMLST

	EC1	EC2	EC3	EC4	EC5	EC6	EC7	EC8	EC9	EC10	EC11	EC12	EC13
EC1	0	0	0	0	0	0	2051	2051	1369	2318	2324	2288	2286
EC2	0	0	0	0	0	0	2053	2053	1370	2320	2326	2290	2288
EC3	0	0	0	0	0	0	2050	2050	1367	2317	2323	2287	2285
EC4	0	0	0	0	0	0	2051	2051	1368	2318	2324	2288	2286
EC5	0	0	0	0	0	0	2053	2053	1370	2320	2326	2290	2288
EC6	0	0	0	0	0	0	2053	2053	1370	2320	2326	2290	2288
EC7	2051	2053	2050	2051	2053	2053	0	1	2021	2338	2344	2304	2302
EC8	2051	2053	2050	2051	2053	2053	1	0	2021	2338	2344	2304	2302
EC9	1369	1370	1367	1368	1370	1370	2021	2021	0	2273	2279	2244	2242
EC10	2318	2320	2317	2318	2320	2320	2338	2338	2273	0	1	2363	2361
EC11	2324	2326	2323	2324	2326	2326	2344	2344	2279	1	0	2369	2367
EC12	2288	2290	2287	2288	2290	2290	2304	2304	2244	2363	2369	0	7
EC13	2286	2288	2285	2286	2288	2288	2302	2302	2242	2361	2367	7	0

Table S3. Overview of resistance genes and phenotype of the fourteen IncI1 pST12 and bla_{CMY-2} containing isolates.

Isolate no.	Species	MultilocusST[a]	Resistance genes	Piperacillin-Tazobactam MIC	Cefotaxime/Ceftriaxone MIC	Ceftazidime MIC	Cefoxitin MIC	Accession no.[b]
**EC1**	*E. coli*	ST665	*bla_{CMY-2}, $bla_{TEM-1b,}$ aada1, strA, strB, sul1, sul2, dfra1*	8 mg/L	4 mg/L	32 mg/L	≥64 mg/L	ERS4591617
**EC2**	*E. coli*	ST665	*bla_{CMY-2}, $bla_{TEM-1b,}$ aada1, strA, strB, sul1, sul2, dfra1*	8 mg/L	8 mg/L	32 mg/L	≥64 mg/L	ERS4591618
**EC3**	*E. coli*	ST665	*bla_{CMY-2}, $bla_{TEM-1b,}$ aada1, strA, strB, sul1, sul2, dfra1*	8 mg/L	4 mg/L	32 mg/L	≥64 mg/L	ERS4591619
**EC4**	*E. coli*	ST665	*bla_{CMY-2}aada1, strA, strB, sul1, sul2, dfra1*	8 mg/L	8 mg/L	32 mg/L	≥64 mg/L	ERS4591620
**EC5**	*E. coli*	ST665	*bla_{CMY-2}, $bla_{TEM-1b,}$ aada1, strA, strB, sul1, sul2, dfra1*	8 mg/L	4 mg/L	32 mg/L	≥64 mg/L	ERS4591621
**EC6**	*E. coli*	ST665	*bla_{CMY-2}, $bla_{TEM-1b,}$ aada1, strA, strB, sul1, sul2, dfra1*	8 mg/L	4 mg/L	32 mg/L	≥64 mg/L	ERS4591622
**EC7**	*E. coli*	ST86	*bla_{CMY-2}, $bla_{TEM-1b,}$, strA, strB, sul2, dfra1*	≤4 mg/L	4 mg/L	8 mg/L	32 mg/L	ERS4591623
**EC8**	*E. coli*	ST86	*bla_{CMY-2}, $bla_{TEM-1b,}$ strA, strB, sul2, dfra1*	≤4 mg/L	8 mg/L	8 mg/L	32 mg/L	ERS4591624
**EC9**	*E. coli*	ST6856	*bla_{CMY-2}, $bla_{TEM-1b,}$*	8 mg/L	8 mg/L	32 mg/L	≥64 mg/L	ERS4591625
**EC10**	*E. coli*	ST131	*bla_{CMY-2}, $bla_{TEM-1b,}$ QnrS1, aac(6')-Ib-cr, aadA16-like[c], ARR-3, sul1, dfrA27, tet(A)-like[c]*	≥128 mg/L	≥64 mg/L	≥64 mg/L	≥64 mg/L	ERS4591626
**EC11**	*E. coli*	ST131	*bla_{CMY-2}, $bla_{TEM-1b,}$ QnrS1*	8 mg/L	8 mg/L	16 mg/L	≥64 mg/L	ERS4591627
**EC12**	*E. coli*	ST973	*bla_{CMY-2}*	8 mg/L	>64 mg/L	32 mg/L	≥64 mg/L	ERS4591628
**EC13**	*E. coli*	ST973	*bla_{CMY-2}*	8 mg/L	8 mg/L	32 mg/L	≥64 mg/L	ERS4591629
**SE1**	*Salmonella enteritidis*	n.a	*bla_{CMY-2}, $bla_{TEM-1b,}$ aac(6')-Iaa-like, rmtB, catA2-like[c], tet(A)-like[c]*	8 mg/L	>4 mg/L	>16 mg/L	>16 mg/L	ERS4591630

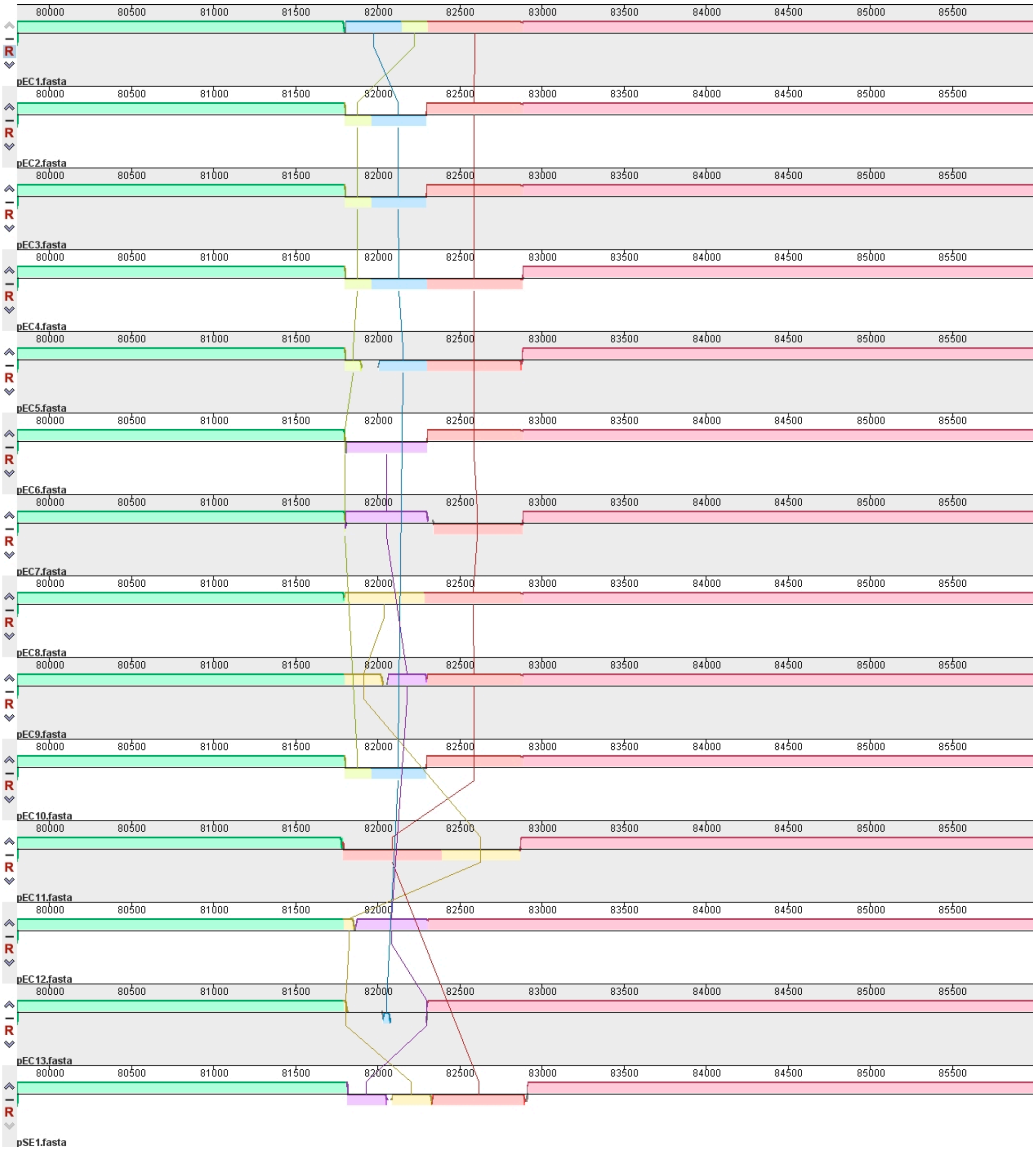

Figure S1. Hypervariable region.
